# Modeling large populations of spiking neurons with a universal population density solver

**DOI:** 10.1186/1471-2202-14-S1-P90

**Published:** 2013-07-08

**Authors:** Marc de Kamps

**Affiliations:** 1 School of Computing, University of Leeds, Leeds, LS2 9JT, UK

## 

Population density techniques are efficient simulation techniques for modeling large homogeneous populations of neurons. When individual spikes are not important, but instead population statistics such as firing rate are relevant, these techniques can replace direct simulations of large groups of point model neurons. The key concept of these methods is a density function that is defined over the state variables of a model neuron, representing how likely a neuron of the population is to be in a given state. The evolution of the density function is given by a partial differential equation that is (related to) a Fokker-Planck equation. The computational gains can be considerable: a second of simulation time of an infinitely large population can be modeled in 0.3 s real time, without requiring post-processing to calculate population statistics. These techniques have been mainly used for leaky-integrate-and-fire neurons, although some applications model conductance-based models with first order synaptic kinetics [1].

In [2] a numerical method, based on the method of characteristics, was presented that is manifestly stable and very efficient; in particular it is not restricted to the diffusion limit, and therefore can handle large synaptic efficacies. In [3] it was demonstrated that the method also models leaky-integrate-and-fire neurons (LIF) in the diffusion limit and reproduces analytic results, demonstrating that it also approximates the solutions of Fokker-Planck equations. Although the method of [2] can in principle be applied to any neuronal model, the representation of the density profile depends critically on the topology of the single neuron model, and spiking models such quadratic-integrate-and-fire (QIF) neurons so far appeared to be non-practical.

Recently [4], it was found that the density can be represented very elegantly for neuronal models that spike periodically, e.g. for QIF neurons with positive input parameter. Moreover, many models, including LIF can be rendered 'spiking' by adding a DC current. By subtracting this DC current from the synaptic input, one can represent the density for these models. The solver is therefore independent of the particular neuronal model, and in that sense universal.

I will present applications of both LIF and QIF neurons. For both model neurons I will show that the method accurately solves the Fokker-Planck equations emerging from the diffusion equation, but remains accurate and efficient when the diffusion limit breaks down. The implication is that this method can solve a large class of (fractional) Fokker-Planck equations. I will show that surprising neural coding strategies are possible for populations of QIF neurons. A recently created MPI-based framework allows efficient network simulations using these techniques [5].
